# A Heterologous V-01 or Variant-Matched Bivalent V-01D-351 Booster following Primary Series of Inactivated Vaccine Enhances the Neutralizing Capacity against SARS-CoV-2 Delta and Omicron Strains

**DOI:** 10.3390/jcm11144164

**Published:** 2022-07-18

**Authors:** Zhiren Zhang, Qiaren He, Wei Zhao, Yong Li, Jiaming Yang, Zhenxiang Hu, Xi Chen, Hua Peng, Yang-Xin Fu, Long Chen, Ligong Lu

**Affiliations:** 1 Zhuhai Institute of Translational Medicine, Zhuhai People’s Hospital, Zhuhai Hospital Affiliated with Jinan University, Zhuhai 519000, China; zhangzhiren2006@163.com (Z.Z.); zhaoweismu@foxmail.com (W.Z.); lorry5160@163.com (Y.L.); 2 The Outpatient Department, Shaoguan Hospital of Traditional Chinese Medicine, Shaoguan 512026, China; 18666613339@163.com; 3 Livzon Bio Inc., Zhuhai 519045, China; yangjiaming@livzon.cn (J.Y.); huzhenxiang@livzon.cn (Z.H.); chenxi01@livzon.cn (X.C.); 4 Key Laboratory of Infection and Immunity, Institute of Biophysics, Chinese Academy of Sciences, Beijing 100101, China; hpeng@moon.ibp.ac.cn; 5 Department of Basic Medical Sciences, School of Medicine, Tsinghua University, Beijing 100084, China; yangxinfu@tsinghua.edu.cn

**Keywords:** V-01, bivalent V-01D-351, inactivated vaccine booster

## Abstract

Immune escape of emerging SARS-CoV-2 variants of concern (VOCs) and waning immunity over time following the primary series suggest the importance and necessity of booster shot of COVID-19 vaccines. With the aim to preliminarily evaluate the potential of heterologous boosting, we conducted two pilot studies to evaluate the safety and immunogenicity of the V-01 or a bivalent V-01D-351 (targeting Delta and Beta strain) booster after 5–7 months of the primary series of inactivated COVID-9 vaccine (ICV). A total of 77 participants were enrolled, with 20 participants in the V-01D-351 booster study, and 27, 30 participants in the age stratified participants of V-01 booster study. The safety results showed that V-01 or V-01D-351 was safe and well-tolerated as a heterologous booster shot, with overall adverse reactions predominantly being absent or mild in severity. The immunogenicity results showed that the heterologous prime–boost immunization with V-01 or bivalent V-01D-351 booster induced stronger humoral immune response as compared with the homologous booster with ICV. In particular, V-01D-351 booster showed the highest pseudovirus neutralizing antibody titers against prototype SARS-CoV-2, Delta and Omicron BA.1 strains at day 14 post boosting, with GMTs 22.7, 18.3, 14.3 times higher than ICV booster, 6.2, 6.1, 3.8 times higher than V-01 booster (10 μg), and 5.2, 3.8, 3.5 times higher than V-01 booster (25 μg), respectively. The heterologous V-01 booster also achieved a favorable safety and immunogenicity profile in older participants. Our study has provided evidence for a flexible roll-out of heterologous boosters and referential approaches for variant-specific vaccine boosters, with rationally conserved but diversified epitopes relative to primary series, to build herd immunity against the ongoing pandemic.

## 1. Introduction

The COVID-19 pandemic continues to surge worldwide, occurring in several waves contributed by the emergence of different variants, with Omicron being the dominant strain circulating in most countries. The Omicron strains, such as BA.1, BA.4, and BA.5, have remarkable growth advantages over ancestral VOCs, such as Beta, Delta, etc., featured by over 30 mutations in spike protein and 15 mutations in its receptor-binding domain (RBD). The newly characteristic mutation at the L452 site in BA.2.12.1 and BA.4, BA.5 lineages has resulted in further immune evasion and elevated transmissibility [1]. The primary series of approved vaccines based on a prototype SARS-CoV-2 exhibited remarkably reduced protective efficacy against Omicron compared to the parental virus strain, as suggested by the recent reports on neutralization capacity [2,3,4] and real-world vaccine effectiveness against symptomatic disease [5,6,7].

Concerns over immune escape of emerging SARS-CoV-2 variants and waning neutralizing antibodies level over time suggest the necessity of the booster shot of COVID-19 vaccines [8]. Additionally, the roll-out of the third or even fourth dose boosters [9,10] has been deployed in appropriate populations in some countries to tackle the ongoing COVID-19 pandemic. Heterologous prime–boost immunization (mix-and-match strategy) has been substantiated as a promising immune strategy because it elicits higher antibody titers and broader cross-neutralizing activity against the emerging VOCs than a homologous booster, paving the way to accelerate vaccination campaigns worldwide [8,11]. While Omicron showed extensive but incomplete escape from the mRNA vaccine-elicited neutralization [2], the mRNA vaccine boosters substantially increased the serum neutralizing activity against Omicron [12,13]. Two candidates of inactivated COVID-19 vaccines: CoronaVac (Sinovac) and BBIBP-CorV (Sinopharm), which gained WHO recommendation on Emergency Use Listing (EUL), have been widely applied for SARS-CoV-2 prevention globally. The neutralizing GMTs against prototype SARS-CoV-2 dramatically declined at 4~8 months post the primary two-dose series, only slightly detectable or below the limit of detection (LOD) [14]. Many countries have adopted a heterologous booster strategy after the primary series of ICV vaccination. A study from the Dominican Republic reported that BNT162b2 booster following a prior two-dose CoronaVac regimen resulted in a 1.4-fold increase in neutralization activity against Omicron compared to the two-dose BNT162b2 regimen [15]. A heterologous BNT162b2 or mRNA-1273 booster markedly increased the humoral and cellular immunity against prototype strain or major VOCs, including Delta and Omicron, after two-dose primary series of ICV [16]. A study from Chile [17] elucidated that heterologous boosters (BNT162b2 or AZD1222) showed higher vaccine effectiveness than a homologous booster after primary series of CoronaVac. 

The recombinant fusion protein vaccine V-01 was designed to enhance antigen processing and presentation by targeting and activating dendritic cells to enhance helper T cell response. In V-01, RBD is armed with an interferon-α at the N-terminus and dimerized by human IgG1 Fc at the C-terminus, with the further addition of a pan HLA-DR-binding epitope (IFN-PADRE-RBD-Fc dimer) to enhance the immune response [18]. Previously, V-01 has shown a favorable safety and immunogenicity profile in phase I and II trials [19,20] and is currently at the stage of two pivotal, international multi-center phase III efficacy trials for primary series and booster after two-dose ICV. In response to emerging variants, a bivalent vaccine V-01D-351, containing major neutralizing epitopes of Beta and Delta, was developed with a hypothesis of providing cross-protection against circulating VOCs. Herein, we investigated whether booster shots of V-01 or variant-specific bivalent V-01D-351 after primary series of ICV, the most widely used vaccine, can produce higher, broader, and more durable viral neutralization activity.

## 2. Materials and Methods

### 2.1. Study Design and Participants

We conducted two pilot studies: a randomized, double-blind, positive control study at the Zhuhai Peoples’ Hospital and an open-label, single-arm study at Shaoguan Hospital of Chinese Medicine. All participants have completed the primary series of two-dose inactivated vaccines (BBIBP-CorV or CoronaVac) in the past 5–7 months; voluntarily consented to participate in this study; agreed to take effective and acceptable contraceptive methods from signing the informed consent form to 3 months after booster vaccination; declared no history of contact with confirmed, asymptomatic, or suspected COVID-19 cases, no history of contact with the individuals from or history of travel in high- and medium-risk epidemic areas. Exclusion criteria were: confirmed COVID-19 cases, or history of previous COVID-19 infection; history of severe allergy to any vaccine or any components of V-01; suspected or diagnosed fever within 72 h before enrollment, or the axillary body temperature ≥ 37.3 °C on the day of enrollment; participants with acute diseases, acute attacks of chronic diseases, or uncontrolled severe chronic diseases; history of congenital or acquired immunodeficiency or autoimmune diseases; pregnant or lactating females, or those who plan to become pregnant within 3 months after the booster dose; receipt of immunoglobulin and/or any blood products within 3 months prior to booster immunization, or with the plan to use such product within 6 months after booster, and any other conditions that, in the opinion of the investigator, might interfere with the assessment of safety and immunogenicity outcomes, or pose additional risks to participants.

The written informed consent form of each participant was obtained at the very beginning of the study. The trials were approved by the Institutional Review Board of the Zhuhai Peoples’ Hospital and Shaoguan Hospital of Chinese Medicine, registered on ClinicalTrials.gov (NCT05238649 accessed on 14 February 2022, and NCT05273528 accessed on 10 March 2022), and conducted in compliance with the Declaration of Helsinki and Good Clinical Practice.

### 2.2. Randomization and Masking

Blinding and masking were not applicable in the single-armed study NCT05273528. Each eligible participant was assigned a unique study number based on the sequential enrollment order. Despite the open-label design, the laboratory staffs were all masked to the blood samples for immunogenicity assessment.

In study NCT05238649, the SAS statistical software, version 9.4 or above, was used to generate a blind table for randomization of subjects and vaccines with a randomized block design. Age stratified participants (18–59 years, ≥60 years) were randomly assigned (1:1:1) to receive the 10 μg V-01, 25 μg V-01, or inactivated vaccine (CoronaVac, Sinovac, Beijing, China). Investigators assigned random numbers to eligible participants according to the order of enrollment. Investigational vaccines for the booster were obtained and administered in line with the random numbers. The randomization statistician was not allowed to participate in other processes of this trial and should not disclose the blinding code to other persons participating in this clinical trial. The participants, investigators, and laboratory staffs were all masked to group allocation during the trial. 

### 2.3. Procedures

The trial flow for the two studies is illustrated in Supplementary Figure S1. A total of 20 eligible participants were recruited in the trial NCT05273528 to receive V-01D-351. Additionally, a total of 57 eligible participants were recruited in trial NCT05238649, with 8, 10, 9 participants in younger adult group (aged 18–59 years) and 10, 10, 10 participants in older adult group (≥60 years) to receive 10 μg V-01, 25 μg V-01, or ICV, respectively.

Screening and vaccination: During the screening period, participants reviewed and completed the signature of ICF, then demographic information, previous and current medical (including allergic) history, and medication (including vaccination) history were collected. The appropriate screening tests were performed to determine the inclusion and exclusion criteria. After screening for eligibility, each participant was assigned a unique study identifier number (NCT05273528) by the sequence of enrollment or randomization number (NCT05238649), then received one booster dose (10 μg + 10 μg bivalent V-01D-351 in study NCT05273528, 10, 25 μg V-01 or ICV in study NCT05238649) intramuscularly in the deltoid muscle of the upper arm. All participants have immunized with the primary two-dose of inactivated vaccine. The investigational vaccine information is described in Table 1.

Safety assessments: All participants must stay on the vaccination site for at least 30 min to observe immediate adverse events (AEs) following the booster immunization. Afterward, participants were trained to fill in the diary card to record the AEs experienced (including solicited local/systemic AE and non-solicited AE), body temperature, and concomitant medications in the subsequent 7 days. On day 8, diary cards were collected and reviewed by the investigator, while contact cards were distributed to participants for documenting AEs 8–28 days after the booster. Solicited local/systemic AEs, categorization, and grading of AEs have been previously specified in our phase I/II trial [19,20].

Immunogenicity assessments: We collected blood samples to determine neutralizing antibody titers against the prototype SARS-CoV-2 and emerging VOCs before and on days 7, 14, 28, and 90 after the booster. Serum samples for humoral immune response underwent cold-chain transfer to the testing laboratory and were stored at −20 °C or below until ready for use. The VSV-based pseudovirus neutralizing assays (Supplementary Methods) against the prototype SARS-CoV-2, Delta, and Omicron BA.1 strain was conducted by t Livzon Bio Inc., Zhuhai, China.

### 2.4. Study Objectives and Outcomes

The two booster studies primarily evaluated the immunogenicity and secondarily assessed the safety of the V-01 or V-01D-351 booster following the two-dose primary inactivated vaccine. The immunogenicity outcomes were geometric mean titer (GMT), geometric mean fold rises (GMFR) against the prototype strain, and VOCs (Delta and Omicron BA.1) after the booster immunization. The safety outcomes were the counts and percentages of AEs (AEs experienced within 30 min, solicited local/systemic and unsolicited AEs within 0–7 days, and unsolicited AEs within 8–28 days after boosting, severe adverse events (SAEs) and adverse of special interest (AESIs) within 90 days). 

### 2.5. Statistical Analysis

The sample size of the two pilot studies was not determined on the basis of a formal statistical hypothesis. The immunogenicity analysis was performed in a boost Per Protocol Set (bPPS), including participants who had completed the booster immunization and had completed predefined blood samplings with available antibody results. The GMTs against prototype SARS-CoV-2 and VOCs after booster immunization with Clopper–Pearson 95% CIs were calculated. Additionally, the GMFR at each time point after booster immunization was statistically described. The safety analysis was performed in a boost Safety Set (bSS), including all participants who received the booster dose. We present counts and percentages of AEs, including overall AEs (solicited and unsolicited AEs), AEs related to vaccination (adverse reactions), AEs graded as grade 3 or worse, AEs leading to participant’s withdrawal, SAEs and AESIs within 90 days after booster. We used the χ² or Fisher’s exact test to analyze categorical data and the t-test to compare log-transformed antibody titers between groups. Statistical analyses were conducted using SAS 9.4 (SAS Institute Inc., San Diego, CA, USA), then data presentation was performed using GraphPad Prism 8.0 (GraphPad Software Inc., San Diego, CA, USA).

## 3. Results

### 3.1. Baseline Characteristics

In study NCT05238649, demographic characteristics (Supplementary Table S1) were comparable across groups, with a mean age of 35.8, 34.0, 39.3 years, a mean prime–boost interval of 162.2, 166, 165.2 days in younger adults, and a mean age of 64.0, 66.5, 67.0 years, a mean prime–boost interval of 161, 166.7, 167.3 days in older adults for the 10 μg V-01 booster, 25 μg V-01 booster, and ICV booster group, respectively. In study NCT05273528, the mean age was 30.7 years with a mean prime–boost interval of 201.6 days. In all groups of both studies, the baseline neutralizing antibody titers to prototype pseudovirus was undetectable or just above the LOD. Both males and females were enrolled in both studies, and a relatively balanced sex ratio was shown.

### 3.2. Safety

V-01 or V-01D-351 was safe and well-tolerated when applied as a heterologous booster shot following the primary series of inactivated vaccines. The overall adverse reactions were absent or mild in severity (Table S2) within 28 days after the booster, and no vaccination-related SAEs or AESIs were observed within 90 days after booster vaccination. The incidence of adverse reactions was comparable across groups, which was 16.7% (3/18), 20.0% (4/20), and 10.5% (2/19) in the 10 μg V-01, 25 μg V-01, and ICV booster groups, respectively, presenting a similar safety profile relative to ICV. In both studies, the most common solicited adverse reaction was injection-site pain, accounting for 15.8% (6/38) and 45% (9/20) following V-01, V-01D-351 booster, which was transient and relived without any treatment.

### 3.3. Immunogenicity

**Figure 1:**
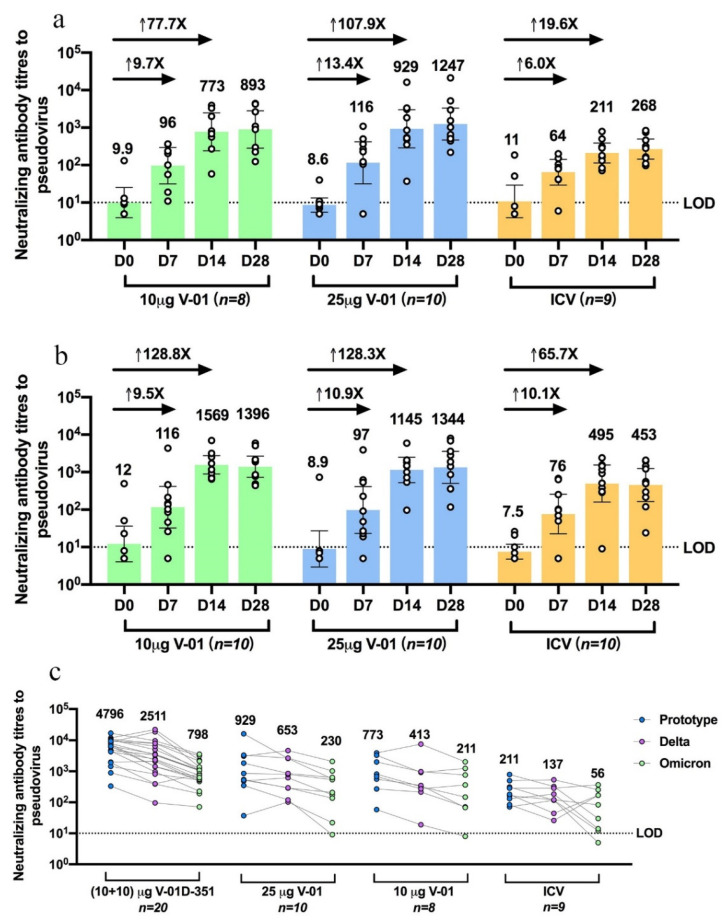
Humoral immune response against prototype SARS-CoV-2, Delta, and Omicron BA.1 strain after V-01 or variant-matched bivalent V-01D-351 booster following primary series of inactivated vaccine. Pseudovirus neutralizing antibody titers against prototype SARS-CoV-2 were analyzed for sera collected at different times (7, 14, 28 days) following a 10μg V-01, 25 μg V-01, or inactivated vaccine booster in younger adults aged 18–59 years (**a**) or older adults aged ≥60 years (**b**). These participants had been primed with two-dose inactivated vaccine 5–7 months earlier. (**c**) Pseudovirus neutralizing antibody titers against prototype SARS-CoV-2, Delta, and Omicron BA.1 strains were analyzed and compared for sera collected from subjects at 14 days following booster of V-01D-351 (individuals in study NCT05273528), 25 μg V-01, 10 μg V-01, or inactivated vaccines (younger adults in study NCT05238649) who completed primary series of inactivated vaccine 5–7 months ago. LOD, the limit of detection.

The V-01 booster elicited a striking increase in the humoral immune response in participants primed with primary two-dose series of ICV both in younger and older adults. Compared with an ICV booster, the neutralizing antibody titers increased more intensively after a V-01 booster (Figure 1a,b) at day 14 relative to baseline, with GMTs of 773 (95%CI: 241–2478) versus 9.9 (3.9–25) in 10 μg V-01, 929 (288–2994) versus 8.6 (5.6–13) in 25 μg V-01, and 211 (114–388) versus 11 (3.9–29) in ICV booster in younger adults; 1569 (893–2758) versus 12 (4.1–36) in 10 μg V-01, 1145 (525–2495) versus 8.9 (2.9–27) in 25 μg V-01, and 495 (159–1544) versus 7.5 (4.8–12) in ICV booster in older adults. On day 14 after the booster immunization, GMFRs were considerably higher in 10 μg, 25 μg V-01 boosters relative to ICV boosters, which were 77.7, 107.9 versus 19.6 times baseline in younger adults, and 128.8, 128.3 versus 65.7 of baseline in older adults. In comparison to younger adults, it is noteworthy that the heterologous 10 and 25 μg V-01 booster showed a favorable immunogenicity profile in older participants, generally with a higher risk for developing severe diseases in the VOCs-circulating COVID-19 pandemic.

**Figure 2:**
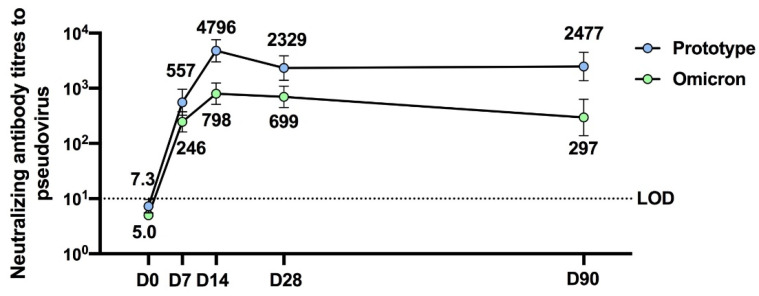
Three-month neutralizing antibody durability against prototype SARS-CoV-2 and Omicron BA.1 strain after the bivalent V-01D-351 booster following primary series of inactivated vaccines (*n* = 20).

The V-01D-351 booster retained potent immunogenicity against the prototype strain and elicited robust cross-neutralizing capacity against Delta and Omicron BA.1. As shown in Figure 1c, younger adults boosted with V-01D-351 tended to exhibit higher neutralization of prototype strain (*n* = 20, 4796: 3013–7633), followed by 25 μg (*n* = 10, 929: 288–2994) and 10 μg V-01 (*n* = 8773: 241–2478), compared with ICV (*n* = 9211: 114–388) at 14 days after the booster. The V-01D-351 group also showed higher neutralization against Delta and Omicron BA.1, followed by 10 μg, 25 μg V-01 compared with ICV booster on day 14, with GMT of 2511 (1325–4756), 653 (255–1671), 413 (100–1700) versus 137 (66–287) for Delta, and 798 (510–1247), 230 (680–775), 211 (46–978) versus 56 (17–183) for Omicron BA.1, respectively. Additionally, the V-01D-351 booster showed slowly waning humoral responses against prototype strain and Omicron BA.1 in 90-day follow-ups. As shown in Figure 2, the V-01D-351 booster induced a substantial increase on day 7, peaked on day 14, and underwent a slight decline from day 28 to day 90, with GMTs of 557 (324–958), 4796 (3013–7633), 2329 (1400–3873), 2477 (1370–4477) against prototype strain, and 246 (162–375), 798 (510–1247), 699 (448–1093), 297 (139–634) against Omicron BA.1 at day 7, 14, 28 and 90 after the booster, respectively. Therefore, the V-01D-351 booster showed the highest pseudovirus neutralizing antibody titers against prototype SARS-CoV-2, Delta, and Omicron BA.1 strains at day 14 post boosting, with GTMs 22.7, 18.3, and 14.3 times higher than ICV booster, 6.2, 6.1, 3.8 times higher than V-01 booster (10 μg), and 5.2, 3.8, 3.5 times higher than V-01 booster (25 μg), respectively.

## 4. Discussion

The preliminary results from the two studies indicated that a heterologous V-01 and bivalent V-01D-351 booster following primary series of ICV enhanced the neutralizing antibody response against prototype SARS-CoV-2 and expanded the breadth of humoral responses to emerging VOCs. Albeit the V-01 was not designed against the VOCs, the immune response induced by a V-01 booster was satisfactory as a heterologous booster. The serum GMTs against the Delta strain on day 14 after the V-01 booster were significantly higher than that against the prototype strain after the ICV booster. The GMTs against the Omicron BA.1 strain on day 14 post the V-01 booster were equivalent to that against the prototype strain after the ICV booster, suggesting the comparable vaccine effectiveness against the VOCs after the V-01 booster versus effectiveness against the prototype strain after the primary series. Individuals boosted with V-01 showed preserved neutralization against Omicron BA.1, only 3.7 to 4-fold lower than prototype SARS-CoV-2, consistent with a 4–6-fold reduction in a study reporting mRNA booster following standard immunization [13]. The antibody response was observed to be high in the older population probably due to the following possible reasons: (1) a small sample size in each group; (2) different immune intervals between two-dose primary series of inactivated vaccines, with an average of 29.6 days versus 46.7 days in the older and younger adult group, respectively. (3) Distinct vaccination profiles of inactivated vaccines for the primary series (younger adults: 6 participants with CoronaVac and 21 with BBIBP-CorV versus older adults: 17 participants with CoronaVac and 13 with BBIBP-CorV); (4) serum samples from younger and older adults were not analyzed head-to-head.

To date, although several variant-matched vaccines have been developed, few variant-specific COVID-19 vaccines are approved for emergency use due to the following possible reasons: (1) the vaccines based on the prototype strain showed slightly declined but preserved effectiveness against the previously circulating VOCs, including Alpha, Beta, Gamma, and Delta, except Omicron [21,22], some of which were only transiently prevalent; (2) Omicron has shown extensive immune escape and poor cross-neutralization by the prototype or other VOC vaccinated anti-serum [23], particularly for the descendent Omicron subvariants, such as BA.4/BA.5 [24]; (3) there have been different opinions regarding whether the Omicron-specific vaccine should be based on the Omicron BA.1 or descendent subvariants such as BA.4/5, and it takes time for a vaccine from initial designed, to clinical development, to approval and scale-up production. Thus, it is critically important to develop vaccines inducing broader neutralization, or even a pan-sarbecovirus vaccine to protect against uncertain future variants. Theoretically, an appropriately designed chimeric vaccine integrating multiple circulating variants, a mosaic vaccine presenting diverse variant-specific antigens, or a bivalent/multivalent vaccine covering the conserved epitopes of circulating variants could be a promising strategy to mount humoral immune response for the respective variants and broaden cross-neutralizing activity to other circulating variants. Bivalent vaccines have become effective tools to respond to the emerging variants. The bivalent vaccine mRNA-1273.211 targeting the prototype and beta strain induced more potent, durable, and wilder humoral responses against key previously circulating VOCs and VOIs, which is superior to mRNA-1273 regarding titers against some VOIs, but also equivalent to peak titers measured after the primary vaccine series against prototype strain [25,26]. Based on the same strategy, the bivalent vaccine mRNA-1273.214 targeting the prototype and Omicron strain has elicited superior neutralizing antibody response against Omicron and non-inferior response against prototype SARS-CoV-2 compared with mRNA-1273 [27]. In our study, the participants receiving the V-01D-351 booster developed appreciable neutralizing activity against Delta and Omicron BA.1, which was 14 times higher than ICV booster and 3.5–6 times higher than V-01 booster, indicating some shared and conserved neutralizing epitopes by Beta and Delta relative to Omicron BA.1. The V-01, particularly the bivalent V-01D-351, may boost neutralization of variants through a mechanism related to antigenic imprinting and affinity maturation, which can expand the functional breadth of immune memory banks against SARS-CoV-2 mutation and evolution [28,29]. The 90-day immune persistence data has shown that the V-01D-351 booster elicits a durable antibody response even against Omicron BA.1, with a 2.4 times reduction in terms of pseudovirus neutralizing titers against Omicron BA.1 after 3 months, which is comparable to a 5.5 times reduction in S protein binding IgGs at 4–5 months after third dose BNT162b2 [30].

Our study has limitations. Firstly, data should be interpreted with caution since the safety and immunogenicity profiles were concluded based on a relatively small sample size. The phase III booster study of V-01 with a larger sample size can provide further evidence regarding this matter [31]. In addition, analysis of cellular mediated immunity was not reported which was readily able to cross recognize VOCs [32,33] and provide protection from severe outcomes. Additionally, a direct comparison of the results from two independent studies may not be appreciated regarding distinct designs: a randomized control trial versus an open-label trial, even though laboratory staff was masked to the biological samples tested. Finally, the neutralizing antibody responses against the currently dominant BA.2 and its descendent lineages, BA.4 and BA.5 were not presented, although the studies were conducted in Omicron BA.1 circulating periods. The enhanced cross-neutralizing activity might be challenged by immune-relevant mutations at L452 in descendent Omicron lineages. Fortunately, L452R mutation was also identified in the Delta strain, which was included in the bivalent V-01D-351.

## 5. Conclusions

Taken together, the heterologous prime–boost immunization with two-dose ICV followed by V-01 or bivalent V-01D-351 booster is well-tolerated and induces robust neutralizing antibodies against SARS-CoV-2 prototype, Delta, and Omicron. The neutralizing antibody response of the bivalent V-01D-351 booster is durable for at least 90 days post boosting. Our study has provided evidence for a flexible roll-out of heterologous boosters and referential approaches for variant-specific vaccine boosters, with rationally conserved but diversified epitopes relative to primary series, to build herd immunity against the ongoing pandemic.

## Figures and Tables

**Table 1:** Information of the investigational vaccine.

Vaccine Name	Design	RBD Sequence
V-01	IFN-PADRE-RBD-Fc dimer	RBD from prototype strain
V-01D-351		RBD from Beta (K417N, E484K and N501Y) and Delta (L452R and T478K), 1:1 mixture

RBD, receptor-binding domain.

## Data Availability

Not applicable.

## Supplementary Materials

The following supporting information can be downloaded at: https://www.mdpi.com/article/10.3390/jcm11144164/s1, Figure S1: Flow diagram of V-01 booster (left) and V-01D-351 booster (right) trial; Table S1: Baseline characteristic of participants; Table S2: Overall adverse events, solicited local and systemic adverse reactions following booster dose of V-01 stratified by age or V-01D-351 booster.
