# Supplementary material for: A Heterologous V-01 or Variant-Matched Bivalent V-01D-351 Booster following Primary Series of Inactivated Vaccine Enhances the Neutralizing Capacity against SARS-CoV-2 Delta and Omicron Strains
Source: J Clin Med. 2022 Jul 18;11(14):4164. doi: 10.3390/jcm11144164 (PMC9317108; doi:10.3390/jcm11144164)
Supplement: Supplementary file 1 [file jcm-11-04164-s001.zip › jcm-1787330-supplementary.pdf]

Table S1. Baseline characteristic of participants.

**Study**	NCT05238649						NCT05273528
	Younger adults (18-59 years)			Older adults (≥60 years)			Younger adults (18-59 years)
	10 μg V-01 (n = 8)	25 μg V-01 (n = 10)	ICV (n = 9)	10 μg V-01 (n = 10)	25 μg V-01 (n = 10)	ICV (n = 10)	V-01D-351 (n = 20)
Age(years)							
Mean (SD)	35.8 (9.7)	34.0 (6.9)	39.3 (7.9)	65.6 (4.2)	66.4 (2.8)	66.3 (4.5)	30.7(8.3)
Median	32.0	33.0	36.0	64.0	66.5	67.0	29.5
Min, Max	27, 53	24, 42	31, 53	60, 72	62, 71	61, 74	19, 50
Sex (%)							
Male	5 (62.5)	7 (70.0)	6 (66.7）	6 (60.0)	5 (50.0)	5 (50.0)	10（50.0）
Female	3 (37.5)	3 (30.0)	3 (33.3)	4 (40.0)	5 (50.0)	5 (50.0)	10（50.0）
Prime-boost interval (days)							
Mean (SD)	162.2 (6.2)	166 (11.0)	165.2(11.3)	161(10.1)	166.7(18.5)	167.3(17.6)	201.6 (11.1)
Median	162.5	162	162	159	160	159	199.5
Min, Max	154, 174	155, 187	154, 190	151, 186	149,195	151, 193	185, 215
Baseline neutralizing antibody titers to wild-type pseudovirus							
GMT	9.9	8.6	11	12	8.9	7.5	7.3
95%CI	3.9, 25	5.6, 13	3.9, 29	4.1, 36	2.9, 27	4.8, 12	5.5, 9.6

Table S2. Overall adverse events, solicited local and systemic adverse reactions following booster dose of V-01 stratified by age or V-01D-351 booster.

**Study**	NCT05238649						NCT05273528
**Adverse events/reactions**	Younger adults (18-59 years)			Older adults (≥60 years)			Younger adults (18-59 years)
	10μg V-01 (n=8)	25μg V-01 (n=10)	ICV (n=9)	10μg V-01 (n=10)	25μg V-01 (n=10)	ICV (n=10)	V-01D-351 (n=20)
**Overall adverse events within 28 days following the booster dose**							
Any	2(25.0)	5(50.0)	4(44.4)	1(10.0)	3(30.0)	1(10.0)	14(70.0)
Vaccination-related	2(25.0)	2(20.0)	2(22.2)	1(10.0)	2(20.0)	0	10(50.0)
Grade 3 or worse	0	0	0	0	0	0	0
Vaccination related SAEs or AESIs within 28-90 days							
SAEs	0	0	0	0	0	0	0
AESIs	0	0	0	0	0	0	0
**Solicited local adverse reactions**							
Pain	2(25.0)	2(20.0)	2(22.2)	1(10.0)	1(10.0)	0	9(45.0)
Pruritus	0	0	0	0	1(10.0)	0	2(10.0)

Swelling	0	0	0	1(10.0)	0	0	0
Redness	0	0	0	0	1(10.0)	0	1(5.0)
Induration	0	0	0	1(10.0)	1(10.0)	0	0
**Solicited systemic adverse reactions**							
Headache	1(12.5)	0	0	0	0	0	1(5.0)
Fatigue	0	0	1 (11.1)	0	0	0	1(5.0)

Data are presented as cases of adverse events or reactions (%)

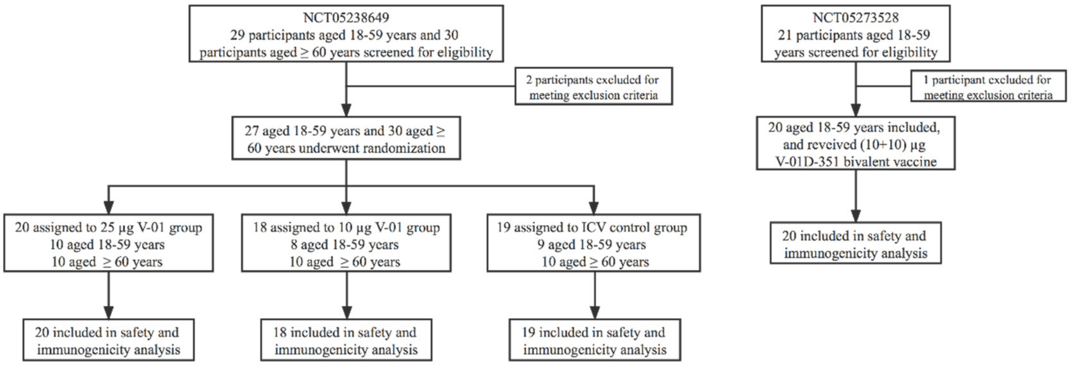

Figure S1. Flow diagram of V-01 booster (left) and V-01D-351 booster (right) trial.

Supplementary Methods

SARS-CoV-2 pseudovirus neutralization assay

Pseudovirus neutralization assay was performed using the VSV-based luciferase reporter SARS-CoV-2 pseudoviruses bearing prototype, delta, and omicron BA.1 variants spike protein, which were provided by Beijing Tiantan Pharmaceutical Biotechnology Development Co. Ltd. The SARS-CoV-2 pseudotyped virus neutralization test began with 3-fold serially diluted heat-inactivated serum samples, which started at 1:5 and then mixed with a certain amount 1000 $TCID_{50}$ of pseudotyped virus for about 1h. Then the mixture was added to 293-hACE2 cells ($2×10^4$ per well). After that, the target cells were incubated for 24 h, and the amount of pseudotyped virus entering the target cells was calculated by detecting the expression of luciferase. The 50% neutralization titer ($pVNT_{50}$) was calculated by GraphPad Prism 8.0 and was defined as the reciprocal of serum dilution at which the relative light units (RUL) were reduced by 50% compared with the virus control wells. Samples with values ≥ 10 were defined as seropositive.
